# Posttransplantation Diabetes Mellitus Among Solid Organ Recipients in a Danish Cohort

**DOI:** 10.3389/ti.2022.10352

**Published:** 2022-04-05

**Authors:** Quenia Dos Santos, Mads Hornum, Cynthia Terrones-Campos, Cornelia Geisler Crone, Neval Ete Wareham, Andreas Soeborg, Allan Rasmussen, Finn Gustafsson, Michael Perch, Soeren Schwartz Soerensen, Jens Lundgren, Bo Feldt-Rasmussen, Joanne Reekie

**Affiliations:** ^1^ Centre of Excellence for Health, Immunity and Infections (CHIP), Rigshospitalet, University of Copenhagen, Copenhagen, Denmark; ^2^ Department of Nephrology, Copenhagen University Hospital Rigshospitalet, Copenhagen, Denmark; ^3^ Department of Clinical Medicine, Faculty of Health and Medical Sciences, University of Copenhagen, Copenhagen, Denmark; ^4^ Department of Surgical Gastroenterology, Rigshospitalet, Copenhagen, Denmark; ^5^ Department of Cardiology, Rigshospitalet, Copenhagen, Denmark

**Keywords:** diabetes mellitus, mortality, transplant, post-transplant diabetes mellitus, solid organ transplant recipient

## Abstract

Post-transplant diabetes mellitus (PTDM) is associated with a higher risk of adverse outcomes. We aimed to describe the proportion of patients with diabetes prior to solid organ transplantation (SOT) and post-transplant diabetes mellitus (PTDM) in three time periods (early-likely PTDM: 0–45 days; 46–365 days and >365 days) post-transplant and to estimate possible risk factors associated with PTDM in each time-period. Additionally, we compared the risk of death and causes of death in patients with diabetes prior to transplant, PTDM, and non-diabetes patients. A total of 959 SOT recipients (heart, lung, liver, and kidney) transplanted at University Hospital of Copenhagen between 2010 and 2015 were included. The highest PTDM incidence was observed at 46–365 days after transplant in all SOT recipients. Age and the Charlson Comorbidity Index (CCI Score) in all time periods were the two most important risk factors for PTDM. Compared to non-diabetes patients, SOT recipients with pre-transplant diabetes and PTDM patients had a higher risk of all-cause mortality death (aHR: 1.77, 95% CI: 1.16–2.69 and aHR: 1.89, 95% CI: 1.17–3.06 respectively). Pre-transplant diabetes and PTDM patients had a higher risk of death due to cardiovascular diseases and cancer, respectively, when compared to non-diabetes patients.

## Graphical Abstract

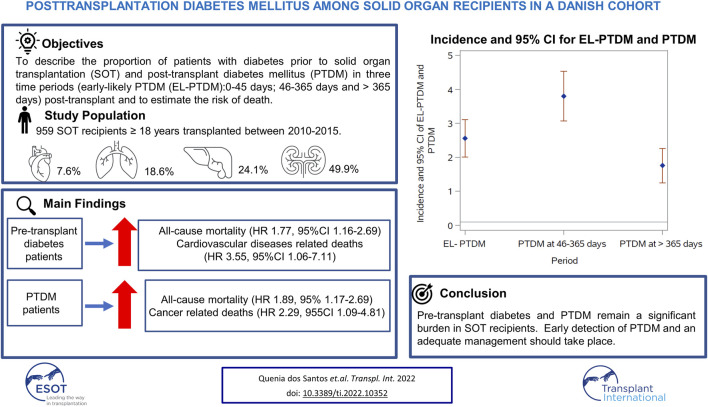

## Introduction

In 2014, the term post-transplantation diabetes mellitus (PTDM) was adopted to refer to newly diagnosed diabetes mellitus in the post transplantation period, irrespective of diagnostic timing or whether diabetes was present but undetected prior to transplantation or not (1). PTDM has been associated with greater mortality and a higher prevalence of infections in Solid Organ Transplant (SOT) recipients (2, 3). PTDM has also been associated with premature or more frequent cardiovascular events among kidney and lung recipients (4, 5) and an increased risk of adverse outcomes in heart recipients, such as cardiovascular morbidity and increased mortality (6–8).

The prevalence of PTDM in the first year after SOT varies by transplanted organ, with previous studies reporting PTDM in 10–20% of patients who have undergone a kidney transplantation (3) and in 20–40% of patients who received other solid organs (9). In addition to transplant type, factors thought to affect the incidence of PTDM include age, body mass index (BMI), race/ethnicity, and the immunosuppression regimen (9). The use of calcineurin inhibitors, especially tacrolimus (10), has been reported to increase the risk of developing PTDM because it can lead to insulin hyposecretion (11–13). Corticosteroids are used as maintenance immunosuppression as well as treatment of rejection and the relationship between this medication and hyperglycemia is well established (2). Therefore, awareness of PTDM risk factors and PTDM management are of importance for post-transplantation care (14).

The International Consensus Meeting on Post Transplant Diabetes Mellitus identified two critical time periods for assessing PTDM (46–365 days and >365 days after transplantation) (1). The consensus meeting also highlighted that due to transient post transplantation hyperglycemia a formal diagnosis of PTDM should not be made in the early time period of 0–45 days post-transplant.

Thus, the aim of this study was to estimate the percentage of SOT recipients with diabetes prior to transplantation and to determine the incidence of and risk factors associated with both early-likely PTDM (EL-PTDM) diagnosed in the 0–45 days post-transplant and PTDM in diagnosed in the two time-periods, 46–365 days and >365 days after transplantation. The inclusion of the EL-PTDM category, was to provide additional information on the transient nature of this period and to determine whether increased monitoring of potential early-likely PTDM patients could be beneficial. In addition, the risk of all cause and cause-specific mortality post-transplant was also assessed and compared in pre-transplant diabetes, those developing PTDM and non-diabetes patients.

## Materials and Methods

### Study Design and Participants

The study cohort included all patients aged ≥18 years who underwent a SOT (heart, liver, lung and kidney) at Rigshospitalet, University Hospital of Copenhagen, a large tertiary transplant center, between January 2010 and December 2015. All SOT patients were prospectively enrolled in the Management of post-Transplant infections in Collaborating Hospitals (MATCH) cohort (15).

For patients with more than one transplantation, only data related to the first transplant after 2010 was assessed**.** Since pancreas transplantation has been demonstrated to accomplish restoration of long-term glucose homeostasis (16, 17), pancreas recipients were excluded.

### Data Sources

Clinical characteristics, sociodemographic and biochemical data were extracted from the MATCH database stored at the Centre of Excellence for Personalized Medicine for Infectious Complications in Immune Deficiency (PERSIMUNE) data warehouse. The data warehouse includes both regional and nationwide data collected prospectively as part of routine care.

Data on prescribed medications including insulin and oral anti-diabetic medication were extracted from the Electronic Prescription Medication (EPM), a database with hospital prescriptions from 2006 to 2016, and the Danish Prescription Database Data (DPDD), a database with outpatient prescriptions from 2004 onwards. Due to a change in systems there was a gap in data from EPM from May 2011 to December 2011. Individual patient data on specific immunosuppressive therapies was not available however, detailed information on the immunosuppressive schemes per transplant type can be found in a previous published article (18) and as a Supplementary Material S1.

Data on admissions and diagnosis were retrieved from the National Patient Registry (LPR) (19) and Sundhedsdatabanken (SDB). LPR was established in 1977 and has national data up to 2016 while SDB has data for patients in the capital region of Denmark from 2008 to 2019. For death, we used data from the Danish Civil Registration System on mortality (20).

In this study we used data on underlying cause of death. Underlying cause of death was defined as the disease or comorbidity leading to the death or directly causing the event classified as the immediate cause of death.

The specific underlying cause of death was obtained in accordance with a modified version of the validated Classification of Death Causes after Transplantation (CLASS) method (21) which includes completion of a Case Record Form for deceased patients and a review and adjudication process involving experts within the field of transplantation (21). The underlying cause of death was selected from 15 pre-defined transplant specific and non-specific categories of death causes, including 267 specific conditions (22). Specific recorded underlying causes of death were further grouped in seven wider categories in order to perform statistical analyses, including deaths due to cardiac or vascular disease, graft failure, graft rejection, infections, cancer, unknown causes and other organ specific diseases (aside from those specified above) and non-organ specific causes. Non-organ specific causes include hemorrhage, alcohol abuse, suicide and other causes.

### Definition of Diabetes

Diabetes was assessed at four time periods, 1) Pre-transplant diabetes, which was defined as a diagnosis of diabetes at any timepoint prior to transplantation. 2) “Early-likely PTDM” (EL-PTDM) assessed in the period 0–45 days post-transplant to help estimate the transient nature of this period and understand whether similar risk factors were identified for EL-PTDM and PTDM. PTDM was then assessed at two time periods post-transplantation according to the periods defined by the International Consensus Meeting on Post Transplant Diabetes Mellitus: 3) 46–365 days, and 4) >365 days post-transplant (1).

Patients were defined as having developed diabetes if they fulfilled at least one of the following criteria during the time period of interest (for all time periods, except before transplant):

• A Hemoglobin A1C test ≥6.5 mmol/L or (1);
• A prescription of antidiabetic medication from either EPM or DPDD (Use of insulin-ATC code A10A, or use of oral antidiabetic medication-ATC code A10B) (23);
• A diagnosis of diabetes (ICD-10 codes: E10, E11, E13) (24).

Patients were classified as having pre-transplant diabetes if they met the above criteria prior to transplantation with the exception of insulin treatment used during hospitalization (from EPM database). Due to high incidence of corticoid-induced hyperglycemia in patients listed for transplantation, patients meeting the definition based only on a record of insulin treatment during hospital admission were not classed as having pre-transplant diabetes.

Patients classified with pre-transplant diabetes remained classified as having diabetes in the entire post-transplantation period. Patients who were not classified as having pre-transplant diabetes could be classified as developing EL-PTDM or PTDM if they met the diabetes definition in the time-period of interest post-transplant. Patients diagnosed with EL-PTDM or PTDM in one period could subsequently return to non-diabetes status in the following time-period if they did not meet the diabetic definition in the new time-period.

During the first 15 days post-transplantation prescription for antidiabetic medication were not included in the definition. A large number of transplant recipients have glucose intolerance and hyperglycemia in the first few weeks post-transplant, detectable in approximately 90% of kidney allograft recipients in the early few weeks after transplant (25, 26). Thus prescription of insulin or oral antidiabetics immediately following transplant and while the patient is hospitalized is high (2), but not an indication of EL-PTDM.

### Approvals

All procedures performed in this study were in accordance with the ethical standards of the 1975 Helsinki Declaration. All relevant approvals were obtained from the Danish National Data Protection Agency (2012-58-0004, RH-2015-67, with I-Suite number: 03787) according to national legislations on retrospective studies. The study was approved by the MATCH steering committee. This work was supported by Danish National Research Foundation (Grant number 126).

### Statistical Analyses

Patient characteristics at transplant were described and compared for those with and without pre-transplant diabetes. Continuous variables, were analyzed using the Wilcoxon test (nonparametric data) and categorical variables, using the χ^2^ test.

The prevalence of diabetes before transplant and incidence of EL-PTDM (0–45 days) and PTDM (46–365 days after transplant and >365 days after transplant) in each of the time periods was calculated among patients alive at the beginning of the time.

Univariable logistic regression analysis was used to evaluate risk factors for developing EL-PTDM and PTDM. Factors that were significant in the univariable analyses (*p*-value < 0.1) were included in the multivariable model. Models were developed separately for each of the three post-transplant time periods. Potential risk factors were selected according to the literature and availability in our dataset (27–30). They included sex; age at transplant; type of transplant; BMI ≥ 25 kg/m^2^ at transplant and the Charlson Comorbidity Index (CCI) (31).

For the CCI (31), two dimensions related to diabetes (presence of diabetes mellitus with and without chronic complications) were excluded from calculation of the index to avoid collinearity issues with our outcome. Therefore 15 dimensions of this index were used.

Survival analysis was used to compare the risk of death in non-diabetes patients to those with pre-transplant diabetes, and those who developed PTDM (>45 days post-transplant). All patients were included in the analysis from day 46 post-transplant (thus individuals who only met the diabetes definition in the EL-PTDM period (0–45 days) were included in the non-diabetes group). Patients were followed until the date of death, a new transplant date, or the end of follow-up, whichever occurred first. For this analysis, the end of the follow-up was set to 31.12.2019 (the last date that cause of death information was available). Diabetes was treated as a time-updated variable, with all patients initially classified as either non-diabetes or pre-transplant diabetes. Patients in the non-diabetes category contributed person-time to that group until such a time as they met our definition for PTDM. They then contributed person-time to the PTDM group from the first date they met our definition for the remainder of the follow-up. Cox proportional hazard models were used to compare the risk of all-cause and cause specific death in the three groups after adjusting for other factors.

As a sensitivity analysis the analysis was re-run using Fine and Grey methodology with deaths not related to the specific cause of interest treated as a competing risk. Categories of causes of death were only assessed if there were more than 20 deaths with that cause.

All data analyses were performed using SAS Studio.

## Results

A total of 959 SOT recipients were included in this study. Two patients with a kidney-pancreas transplant were excluded. The most common transplant type was kidney (479, 50.0%), followed by liver (231, 24.0%), lung (176, 18.0%) and heart (73, 8.0%). Pre-transplant diabetes was observed in 334 (34.8%–95% CI: 31.8–37.9) SOT recipients, with 78.0% meeting our definition in the year prior to transplantation. Of those 334 patients with pre-transplant diabetes, 33.5% (112 patients) met all three diabetes criteria; 7 (2.0%) had a medication prescription and a hemoglobin A1C ≥ 6.5 mmol/L only; 25 (7.5%) had a hemoglobin A1C ≥ 6.5 mmol/L and a diagnosis code only; 30 (9.0%) had a diagnosis code and a medication prescription only; 133 patients (39.9%) with one hemoglobin A1C ≥ 6.5 mmol/L, 23 (6.9%) with a diagnosis code, 4 (1.2%) with a medication prescription.

Table 1 shows the patient characteristics by pre-transplant diabetes status. There was a higher percentage of patients with BMI < 25 among non-diabetes compared to pre-transplant diabetes (64.9%, 95% CI: 59.8–69.7 vs. 35.1%, 95% CI: 30.2–40.1). A higher percentage of pre-transplant diabetes was observed among heart (56.2–95% CI: 44.0–67.7) and kidney transplants (39.9–95% CI: 35.4–44.4) compared to lung (31.8–95% CI: 25.0–39.2) and liver (19.9–95% CI: 14.9–25.6) (*p* = 0.001). The median age at transplant was also higher in those with pre-transplant diabetes compared to non-diabetes: 52.8 years (95% CI: 44.7–60.2) vs. 48.9 years (95% CI: 39.6–55.0).

**TABLE 1:** Characteristics of non-diabetes and diabetes patients at baseline.

Characteristics at baseline	Non-diabetes baseline (*n* = 625)	Pre-transplant diabetes (*n* = 334)	*p*-Value
Type of Transplant-N (%)			
Kidney	288 (60.1)	191 (39.9)	0.001
Liver	185 (80.1)	46 (19.9)	
Lung	120 (68.2)	56 (31.8)	
Heart	32 (43.8)	41 (56.2)	
Sex-N (%)			
Male	366 (63.2)	213 (36.8)	0.11
Female	259 (68.2)	121 (31.8)	
BMI categories-N (%)			
BMI < 25.0	244 (64.9)	132 (35.1)	0.006
BMI ≥ 25.0	191 (58.6)	135 (41.4)	
Missing	190 (72.0)	67 (28.0)	
Age in years (Median & IQR)	48.9 (39.6–55.0)	52.8 (44.7–60.2)	0.002
CCI in points (Median & IQR)	2.0 (2.0–3.0)	2.0 (2.0–3.0)	0.99

**FIGURE 1:**
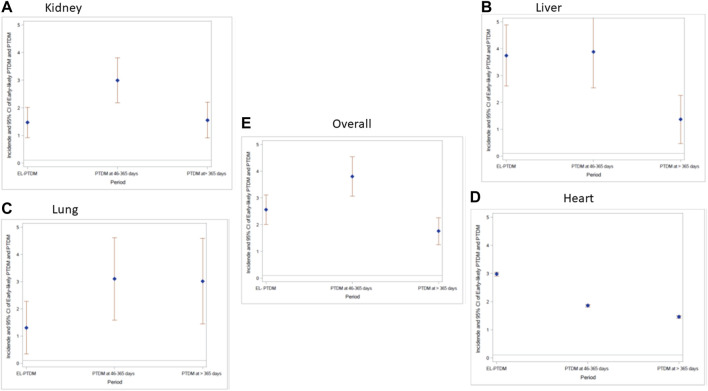
Incidence rate per 100 person-days follow up and 95% Confidence interval of EL-PTDM and PTDM overall and per transplant type at each time period. **(A)** Kidney; **(B)** Liver, **(C)** Lung; **(D)** Heart; **(E)** Overall.

The number and percentage of non-diabetes, pre-transplant diabetes and PTDM overall and per transplant type at each time period is found in Supplementary Material S2. The highest incidence of PTDM was observed at 46–365 days after transplant (IR of 3.80, 95% CI: 3.07–4.53) per 100 PYFU vs. IR of 2.56, 95% CI: 2.01–3.11 for EL-PTDM and IR of 1.76, 95% CI: 1.25–2.26 at >365 days after transplant (Figure 1). Among the 625 SOT recipients with no pre-transplant diabetes, 83 (13.3%) fulfilled the diagnosis criteria for EL- PTDM in the first 45 days post-transplant. Between day 46 and day 365 post-transplant, 171 patients (28.0%), out of 611 patients under follow-up at day 46, met our criteria for PTDM; 104 were new PTDM and 67 had also been diagnosed in the previous time period and 16 PTDM detected in the previous period reverted to non-diabetes. In the late period (>365 days) 143 patients out of 579 still under follow-up after 1 year had PTDM (24.7%) of whom 47 met the criteria for the first time and 96 were already diagnosed as PTDM in one of the previous periods and 62 patients diagnosed with PTDM in the previous period reverted to non-diabetes. The number of patients and the distribution of EL-PTDM and PTDM diagnostic criteria can be found in the Supplementary Material S3.

Risk factors associated with the development of EL-PTDM and PTDM in univariable analysis are shown in Table 2. For the multivariable analyses, older age and a higher CCI score in all time periods remained significantly associated with an increased likelihood of EL-PTDM and PTDM. For the EL-PTDM patients, the adjusted odds ratio (aOR) for age and CCI were (aOR: 1.44 per 10 years older, 95% CI: 1.18–1.75, *p* = 0.0003 and aOR: 1.39, 95% CI: 1.17–1.65, *p* = 0.0002 respectively). At 46–365 days, the estimates for age and CCI score were (aOR: 1.40 per 10 years older, 95% CI: 1.20–1.62, *p* = 0.0001 and aOR: 1.23, 95% CI: 1.06–1.42, *p* = 0.005) and in the later period (aOR: 1.44, 95% CI: 1.22–1.69, *p* = 0.0001 and aOR: 1.23, 95% CI: 1.04–1.44, *p* = 0.01 respectively).

**TABLE 2:** Univariable risk factors for the development of EL-PTDM and PTDM in each time period.

	EL-PTDM		46–365 days after transplant		>365 days after transplant	
	*n* = 625		*n* = 611		*n* = 579	
Variables	OR with 95% CI	*p*-Value	OR with 95% CI	*p*-Value	OR with 95% CI	*p*-Value
Sex						
Male	1 (ref)		1 (ref)		1 (ref)	
Female	0.82 (0.51–1.31)	0.41	0.89 (0.62–1.28)	0.55	1.01 (0.69–1.49)	0.92
Transplant type						
Kidney	1 (ref)		1 (ref)		1 (ref)	
Liver	2.43 (1.46–4.04)	0.006	1.76 (1.18–2.64)	0.005	1.37 (0.88–2.11)	0.15
Lung	0.67 (0.31–1.45)	0.31	0.66 (0.39–1.13)	0.13	0.91 (0.52–1.57)	0.73
Heart	1.91 (0.73–5.01)	0.18	1.01 (0.42–2.33)	0.99	0.81 (0.32–2.08)	0.67
BMI categories						
BMI < 25	1 (ref)		1 (ref)		1 (ref)	
BMI ≥ 25	2.42 (1.42–4.19)	0.001	1.59 (1.05–2.43)	0.02	2.16 (1.38–3.37)	0.007
BMI-missing	1.32 (0.72–2.41)	0.35	0.99 (0.64–1.55)	0.98	1.06 (0.64–1.74)	0.81
Age (each 10 years)	1.43 (1.18–1.74)	0.002	1.39 (1.20–1.62)	0.001	1.44 (1.22–1.69)	0.001
CCI score (per point higher)	1.37 (1.15–1.63)	0.003	1.22 (1.06–1.42)	0.006	1.23 (1.05–1.44)	0.009

A total of 174 patients died during 4,636 person years follow-up (PYFU) (Table 3) (IR 3.75, 95% CI: 3.20–4.31) per 100 PYFU. PTDM patients were found to have a higher risk of death when compared to non-diabetes patients in the univariable analysis (HR: 1.46, 95% CI: 1.03–2.07). This increased risk remained for PTDM and became significant for pre-transplant diabetes patients after adjusting for sex, age (per 10 years), BMI, diabetes groups, CCI and transplant type (aHR of 1.89, 95% CI: 1.17–3.06 and aHR of 1.77, 95% CI: 1.16–2.69 respectively, Table 3).

**TABLE 3:** Number of deaths and incidence rate (95% CI) of death per 100 PYFU and univariable and multivariable Cox models for death per diabetes group.

	N of deaths	Incidence Rate (95% CI)	Univariable^a^		Multivariable^b^		Multivariable^c^	
			HR (95%CI)	*p*-Value	HR (95%CI)	*p*-Value	HR (95%CI)	*p*-Value
Non-diabetes	71	3.14 (2.41–3.87)	1	-	1	-	1	-
Pre-transplant diabetics	64	4.09 (3.09–5.09)	1.32 (0.93–1.88)	0.11	1.35 (0.93–1.98)	0.11	1.77 (1.16–2.69)	0.007
PTDM	39	4.84 (3.32–6.36)	1.46 (1.03–2.07)	0.03	1.53 (0.99–2.39)	0.05	1.89 (1.17–3.06)	0.008

a Univariable model: adjusted by diabetes groups.

b Multivariable model: adjusted for sex, age (per 10 years), BMI, diabetes groups and CCI.

c Multivariable model: adjusted for sex, age (per 10 years), BMI, diabetes groups; CCI, and transplant type.

Table 4 shows the distribution of cause of deaths per diabetes group. In the univariable analysis, when compared to nondiabetes patients, a higher risk of death due to cardiovascular diseases was found and remained after adjustment for other risk factors (aHR 3.55, 95% CI 1.06–11.74). A higher rate of deaths due to cancer was observed in PTDM patients in both univariable and multivariable models (HR of 2.62, 95% CI: 1.22–5.62, *p* = 0.01 and aHR of 2.29, 95% CI: 1.09–4.81, *p* = 0.02, respectively). No other significant differences were found for the remaining causes of death among non-diabetes, pre-transplant diabetes and PTDM.

**TABLE 4:** Number of deaths per cause of death and incidence rate (95% CI) of deaths per 100 PYFU and univariable and multivariable Cox models for death causes per diabetes group.

Causes of death	Diabetes group	N of deaths	Incidence rate (95% CI)	Univariable^a^	Multivariable^b^	HR (95% CI)	*p*-Value
				HR (95% CI)	*p*-Value		
Graft rejection	Non-diabetes	19.0	0.84 (0.46; 1.22)	1	-	1	-
	Pre-transplant diabetes	10.0	0.64 (0.24; 1.03)	0.77 (0.35; 1.65)	0.50	1.53 (0.55; 4.21)	0.40
	PTDM	9.0	1.12 (0.39; 1.85)	1.33 (0.59; 2.99)	2.72	1.50 (0.89; 8.24)	0.08
Infections	Non-diabetes	12.0	0.53 (0.23; 0.83)	1	-	1	-
	Pre-transplant diabetes	9.0	0.57 (0.20; 0.95)	1.08 (0.45; 2.60)	0.85	1.85 (0.74; 4.63)	0.18
	PTDM	6.0	0.74 (0.15; 1.34)	1.40 (0.51; 3.78)	0.50	2.06 (0.67; 6.33)	0.20
Cardiovascular diseases	Non-diabetes	5.0	0.22 (0.03; 0.41)	1	-	1	-
	Pre-transplant diabetes	13.0	0.83 (0.38; 1.28)	3.89 (1.38; 10.95)	0.01	3.55 (1.06; 11.74)	0.03
	PTDM	3.0	0.37 (0.21; 0.79)	1.73 (0.41; 7.28)	0.44	1.57 (0.34; 7.11)	0.55
Cancer	Non-diabetes	14.0	0.62 (0.29; 0.94)	1	-	1	-
	Pre-transplant diabetes	17.0	1.09 (0.57; 1.60)	1.78 (0.87; 3.63)	0.11	1.83 (0.86; 3.93)	0.11
	PTDM	13.0	1.61 (0.74; 2.49)	2.62 (1.22; 5.62)	0.01	2.29 (1.09; 4.81)	0.02

a Univariable model: adjusted by diabetes groups.

b Multivariable model: adjusted for sex, age (per 10 years), BMI, diabetes groups; CCI, and transplant type.

## Discussion

In this study of 959 SOT recipients, over one third fulfilled our diagnosis criteria for diabetes prior to transplantation. The highest proportion was among heart recipients where slightly over half of the patients met our criteria for pre-transplant diabetes. The proportion of non-diabetes patients at transplantation diagnosed with PTDM was also high, with the highest incidence rates observed at 46–365 days post-transplantation (IR of 3.80, 95% CI: 3.07–4.53). Older age and a higher CCI score (at all time periods) were associated with an increased risk of PTDM and similar risk factors were identified for EL-PTDM. A higher incidence rate of all-cause mortality was observed among individuals with diabetes prior to transplantation and PTDM patients. Pre-transplant diabetes and PTDM patients were found to have a higher risk of death due to cardiovascular disease and cancer in both univariable and multivariable analysis.

The characteristics of our patients with pre-transplant diabetes (Table 1) were consistent with previous studies (4, 32–34). We also observed a similar proportion with pre-transplant diabetes (34.8–95% CI: 31.8–37.9), where previous studies have estimated the prevalence to range from 17.5 to 38.0% (4, 32–34). These studies used a variety of different criteria to assess diabetes. Some included oral glucose tolerance test or fasting plasma glucose (35, 36), variables not available in this study or only the combinations of two components (such as two or more positive random glucose or a hemoglobin A1C ≥6.5 prior to transplant) (32), while other studies have relied on self-report diabetes status (34, 37). However, a recent study, using a criteria similar to ours, found high sensitivity (93%) and specificity (98%) when comparing their criteria against patient self-report diabetes status (37), and found the combined criteria better than using diagnosis or medication alone.

The formal diagnosis of PTDM is recommended when the patients are stable on their likely maintenance immunosuppression and in the absence of acute infections (1). In addition, most studies report the percentage of SOT recipients with PTDM at time periods equal or greater than 1 year after transplant. It is well known that an excess in blood glycemia can occur for myriad reasons post-transplantation (immunosuppressive therapy, infections, and other critical conditions), and thus it is important to exclude transient post transplantation hyperglycemia from PTDM diagnosis. Previous studies have reported hyperglycemia in approximately 90% of kidney allograft recipients during the first weeks post-transplant (25, 26). Consequently, in the immediate post-transplant setting, insulin therapy or prescription of a medication for diabetes is generally required to manage postoperative hyperglycemia, especially given the requirement for high-dose immunosuppressants in this setting (2). We split the present analysis into two PTDM time periods (46–365 days and >365 days), but we also reported patients that fulfilled the diabetes criteria in the first 45 days after transplant (EL-PTDM) to increase awareness of the number of transplant recipients that can potentially develop PTDM in the future. Furthermore, it is important to emphasize that the first weeks after transplant are critical periods and efforts should be made in a tentative effort to stabilize the patient. Of a total of 83 patients diagnosed with EL-PTDM in the first 45 days after transplant, 67 (80.7%) remained PTDM in the subsequent period.

The highest incidence of overall PTDM and overall diabetes was detected at 46–365 days. This is in line with the literature, that recommends that a diagnosis of PTDM is generally reserved for the outpatient setting, when the recipient had been discharged from the hospital, is stable, and in the absence of acute infections (1, 2).

The percentage of PTDM at 1 year post-transplant found in the literature ranged from 12 to 45% in liver recipients (32); 4–25% in kidney transplant recipients (34); 4–40% in heart transplant recipients (29, 38); and 5–45% in lung transplant recipients (39-41). This again is in line with our results reported for days 46–365 (liver recipients: 38.1%, heart recipients: 25.8% kidney: 25.7% and lung: 18.8%) (Supplementary Material S2).

The most important risk factors for the development of EL-PTDM and PTDM in this study were age and CCI score (in all time periods), which have been identified previously (27–30, 42). Some immunosuppressive regimens have also been associated with an increased risk of PTDM. This could not be investigated by our study due to limitations in our medication data and the lack of reliable information on the medication dosages.

Pre-transplant diabetes and PTDM patients were found to have a higher all-cause mortality rate and in cause-specific analysis patients with pre-transplant diabetes had a higher risk of death due to cardiovascular diseases when compared to non-diabetes. This is in line with previous studies (2, 3, 27, 30, 43, 44), that also used similar covariates in their analyses (age, gender, BMI, among others). An important study (2) found that PTDM may only reduce short-term survival after liver transplant, while the impact of PTDM on survival after lung transplant is unclear and PTDM after heart transplantation does not affect survival. In our study, PTDM patients, had a higher risk of death due to cancer in the univariable analysis and in the multivariable analysis. This was also observed in a previous study (44) while some other studies did not support this finding (4, 45). It is well known that diabetes mellitus has been widely associated with the increase the risk of malignancy due to the postulated mechanisms including stimulation of insulin-like growth factor-axis and increased cytokines production (46), but it is still uncertain whether the same association can be extrapolated to PTDM patients (44). For the remaining causes of death, no differences were found when comparing pre-transplant diabetes and PTDM to non-diabetes patients.

The limitations of this study should be highlighted. As mentioned previously (25, 26), increases in the glycemia levels are expected in the period right after transplant (25, 26), and it is not common for a patient to receive a diagnostic code for diabetes at 0–45 days after transplant. Additionally, our criteria to define PTDM does not include blood glucose levels as the available data did not discriminate between fasting and non-fasting glucose tests. Thus, it is possible, that the incidence of EL-PTDM could be underestimated particularly in the 0–45 days period. Further, patients with chronic kidney disease may have a lower hemoglobin because of erythropoietin deficit, especially right after transplant. However, the number of EL-PTDM patients diagnosed only based on HbA1C in this time period is low (27.8%) (Supplementary Material S3). One additional limitation is the lack of information about the immunosuppressive medication as previously mentioned. Furthermore, for some cause of deaths the number of events was very low, therefore their results must be interpreted cautiously. Lastly, as data on medication was available only until the December 31, 2016, incidence of PTDM could be underestimated from 2017 until 2019, since for this period it relied on hemoglobin A1c and diabetes diagnosis codes only.

The strengths of this study are to present the PTDM frequency in different time periods and to include different types of solid organs recipients (kidney, liver, lung and heart) as well as to report the number of EL-PTDM patients. An additional strength is that this is the first study that assess post-transplant death between non-diabetes, pre-transplant diabetics and PTDM since most of the published studies combine PTDM and pre-transplant diabetes together or exclude pre-transplant diabetes and present the outcomes only for non-diabetes and PTDM.

In conclusion, this study found that a high proportion of SOT recipients have diabetes prior to transplantation, and that PTDM incidence was highest at 46–365 days after transplant in all transplant recipients. Compared to non-diabetes, pre-transplant diabetics and PTDM patients had a higher mortality rate after transplant. In relation to causes of death, pre-transplant diabetes and PTDM patients had a higher risk of death due to cardiovascular diseases and cancer, respectively, when compared to non-diabetes patients. Pre-transplant diabetes and PTDM remain a significant burden in the SOT population and an early detection of PTDM and an adequate management and treatment of both pre-transplant diabetes and PTDM should take place. For those patients, it is advisable to follow current general practice guidelines for blood glucose goals for both inpatients (47) and outpatients (48). Closer monitoring and frequent (49) follow-up are of the utmost importance to prevent or minimize adverse outcomes in those patients.

## Data Availability

The datasets generated during and/or analyzed during the current study are available from the corresponding author on reasonable request. Requests to access the datasets should be directed to persimune.rigshospitalet@regionh.dk.

## Abbreviations

ATC, anatomical therapeutic chemical; BMI, body mass index; CCI, Charlson comorbidity index; CI, confidence interval; CLASS, classification of death causes after transplantation; DPDD, Danish Prescription Database Data; EPM, electronic prescription medication; ICD, International Classification of Diseases; IR, incidence rate; IRR, incidence rate ratio; LPR, National Patient Registry; MATCH, Management of post-transplant infections in collaborating Hospitals; PERSIMUNE, Personalized Medicine for Infectious Complications in Immune Deficiency; PTDM, post-transplant diabetes mellitus; SDB, Sundhedsdatabanken; PYFU, Person Year Follow-up; SOT, solid organ transplant.

## References

[1] Sharif A Hecking M de Vries APJ Porrini E Hornum M Rasoul-Rockenschaub S Proceedings from an International Consensus Meeting on Posttransplantation Diabetes Mellitus: Recommendations and Future Directions. Am J Transpl (2014) 14(9):1992–2000. 10.1111/ajt.12850 PMC4374739 25307034

[2] Shivaswamy V Boerner B Larsen J. Post-transplant Diabetes Mellitus: Causes, Treatment, and Impact on Outcomes. Endocr Rev (2016) 37(1):37–61. 10.1210/er.2015-1084 26650437 PMC4740345

[3] Kasiske BL Snyder JJ Gilbertson D Matas AJ. Diabetes Mellitus after Kidney Transplantation in the United States. Am J Transpl (2003) 3(2):178–85. 10.1034/j.1600-6143.2003.00010.x 12603213

[4] Eide IA Halden TAS Hartmann A Åsberg A Dahle DO Reisaeter AV Mortality Risk in post-transplantation Diabetes Mellitus Based on Glucose and HbA1c Diagnostic Criteria. Transpl Int (2016) 29(5):568–78. 10.1111/tri.12757 26875590

[5] Seoane-Pillado MT Pita-Fernández S Valdés-Cañedo F Seijo-Bestilleiro R Pértega-Díaz S Fernández-Rivera C Incidence of Cardiovascular Events and Associated Risk Factors in Kidney Transplant Patients: a Competing Risks Survival Analysis. BMC Cardiovasc Disord (2017) 17(1):72. 10.1186/s12872-017-0505-6 28270107 PMC5341360

[6] Cho MS Choi H-I Kim I-O Jung S-H Yun T-J Lee J-W The Clinical Course and Outcomes of post-transplantation Diabetes Mellitus after Heart Transplantation. J Korean Med Sci (2012) 27(12):1460–7. 10.3346/jkms.2012.27.12.1460 23255843 PMC3524423

[7] Kim HJ Jung S-H Kim J-J Yun T-J Kim JB Choo SJ New-Onset Diabetes Mellitus after Heart Transplantation- Incidence, Risk Factors and Impact on Clinical Outcome. Circ J (2017) 81(6):806–14. 10.1253/circj.cj-16-0963 28344200

[8] Martínez-Dolz L Almenar L Martínez-Ortiz L Arnau MA Chamorro C Moro J Predictive Factors for Development of Diabetes Mellitus post-heart Transplant. Transpl Proc (2005) 37(9):4064–6. 10.1016/j.transproceed.2005.09.161 16386627

[9] Jenssen T Hartmann A. Post-transplant Diabetes Mellitus in Patients with Solid Organ Transplants. Nat Rev Endocrinol (2019) 15(3):172–88. 10.1038/s41574-018-0137-7 30622369

[10] Heisel O Heisel R Balshaw R Keown P. New Onset Diabetes Mellitus in Patients Receiving Calcineurin Inhibitors: a Systematic Review and Meta-Analysis. Am J Transpl (2004) 4(4):583–95. 10.1046/j.1600-6143.2003.00372.x 15023151

[11] Santos L Rodrigo E Piñera C Quintella E Ruiz JC Fernández-Fresnedo G New-onset Diabetes after Transplantation: Drug-Related Risk Factors. Transplant Proc (2012) 44(9):2585–7. 10.1016/j.transproceed.2012.09.053 23146462

[12] Shah T Kasravi A Huang E Hayashi R Young B Cho YW Risk Factors for Development of New-Onset Diabetes Mellitus after Kidney Transplantation. Transplantation (2006) 82(12):1673–6. 10.1097/01.tp.0000250756.66348.9a 17198258

[13] Yates CJ Fourlanos S Hjelmesaeth J Colman PG Cohney SJ. New-onset Diabetes after Kidney Transplantation-Changes and Challenges. Am J Transpl (2012) 12(4):820–8. 10.1111/j.1600-6143.2011.03855.x 22123607

[14] Weng LC Chiang YJ Lin MH Hsieh CY Lin SC Wei TY Association between Use of FK506 and Prevalence of post-transplantation Diabetes Mellitus in Kidney Transplant Patients. Transplant Proc (2014) 46(2):529–31. 10.1016/j.transproceed.2013.11.141 24656004

[15] Lodding IP Sengeløv H da Cunha-Bang C Iversen M Rasmussen A Gustafsson F Clinical Application of Variation in Replication Kinetics during Episodes of Post-transplant Cytomegalovirus Infections. EBioMedicine (2015) 2(7):699–705. 10.1016/j.ebiom.2015.05.003 26288842 PMC4534685

[16] Morel P Goetz FC Moudry-Munns K Freier E Sutherland DE. Long-term Glucose Control in Patients with Pancreatic Transplants. Ann Intern Med (1991) 115(9):694–9. 10.7326/0003-4819-115-9-694 1929037

[17] Robertson RP Sutherland DE Lanz KJ. Normoglycemia and Preserved Insulin Secretory reserve in Diabetic Patients 10-18 Years after Pancreas Transplantation. Diabetes (1999) 48(9):1737–40. 10.2337/diabetes.48.9.1737 10480602

[18] Ekenberg C da Cunha-Bang C Lodding IP Sørensen SS Sengeløv H Perch M Evaluation of an Electronic, Patient-Focused Management System Aimed at Preventing Cytomegalovirus Disease Following Solid Organ Transplantation. Transpl Infect Dis (2020) 22(2):e13252. 10.1111/tid.13252 31997565

[19] Lynge E Sandegaard JL Rebolj M. The Danish National Patient Register. Scand J Public Health (2011) 39(7_Suppl. l):30–3. 10.1177/1403494811401482 21775347

[20] Pedersen CB. The Danish Civil Registration System. Scand J Public Health (2011) 39(7 Suppl. l):22–5. 10.1177/1403494810387965 21775345

[21] Wareham NE Da Cunha-Bang C Borges ÁH Ekenberg C Gerstoft J Gustafsson F Classification of Death Causes after Transplantation (CLASS). Medicine (Baltimore) (2018) 97(29):e11564. 10.1097/md.0000000000011564 30024557 PMC6086480

[22] Rigshospitalet UoC. Cause of Death List: Rigshospitalet. Copenhagen, Denmark: University of Copenhagen, Centre of Excellence for Health, Immunity and Infections (CHIP). Available from: https://chip.dk/Portals/0/files/MATCH/CLASS_Cause%20of%20Death%20List.pdf?ver=2018-10-01-095315-583×tamp=1538380416865 (Accessed December 16, 2021).

[23] Methodology WHOCC. Drugs Used in Diabetes. Norway: Norwegian Institute of Public Health. Available from: https://www.whocc.no/atc_ddd_index/?code=a10 (Accessed November 15, 2021).

[24] ICD10Data. Diabetes Mellitus E08-E13 (2022). Available from: ICD10Data.com .

[25] Chakkera HA Weil EJ Castro J Heilman RL Reddy KS Mazur MJ Hyperglycemia during the Immediate Period after Kidney Transplantation. Cjasn (2009) 4(4):853–9. 10.2215/cjn.05471008 19339426 PMC2666437

[26] Hecking M Haidinger M Döller D Werzowa J Tura A Zhang J Early Basal Insulin Therapy Decreases New-Onset Diabetes after Renal Transplantation. Jasn (2012) 23(4):739–49. 10.1681/asn.2011080835 22343119 PMC3312499

[27] Cheng CY Chen CH Wu MF Wu MJ Chen JP Liu YM Risk Factors in and Long-Term Survival of Patients with Post-Transplantation Diabetes Mellitus: A Retrospective Cohort Study. Int J Environ Res Public Health (2020) 17(12), 4581. Available from: http://europepmc.org/abstract/MED/32630562 . https://europepmc.org/articles/PMC7345656 . https://europepmc.org/articles/PMC7345656?pdf=render. 10.3390/ijerph17124581 PMC7345656 32630562

[28] Demirci MS Toz H Yılmaz F Ertilav M Asci G Ozkahya M Risk Factors and Consequences of post-transplant Diabetes Mellitus. Clin Transpl (2010) 24(5):E170–E177. 10.1111/j.1399-0012.2010.01247.x 20384711

[29] Pham PT Sidhu HS Pham PM Pham PC Diabetes Mellitus after Solid Organ Transplantation. In: Feingold KR Anawalt B Boyce A editors. Endotext [Internet]. South Dartmouth (MA): MDText.com, Inc. (2019). Available from: https://www.ncbi.nlm.nih.gov/books/NBK378977/ (Accessed November 28, 2021).

[30] Roccaro GA Goldberg DS Hwang W-T Judy R Thomasson A Kimmel SE Sustained Posttransplantation Diabetes Is Associated with Long-Term Major Cardiovascular Events Following Liver Transplantation. Am J Transpl (2018) 18(1):207–15. 10.1111/ajt.14401 PMC5740009 28640504

[31] Quan H Sundararajan V Halfon P Fong A Burnand B Luthi J-C Coding Algorithms for Defining Comorbidities in ICD-9-CM and ICD-10 Administrative Data. Med Care (2005) 43(11):1130–9. 10.1097/01.mlr.0000182534.19832.83 16224307

[32] Lieber SR Lee RA Jiang Y Reuter C Watkins R Szempruch K The Impact of post-transplant Diabetes Mellitus on Liver Transplant Outcomes. Clin Transpl (2019) 33(6):e13554. 10.1111/ctr.13554 PMC6995642 30927288

[33] Hjelmesaeth J Hartmann A Leivestad T Holdaas H Sagedal S Olstad M The Impact of Early-Diagnosed New-Onset post-transplantation Diabetes Mellitus on Survival and Major Cardiac Events. Kidney Int (2006) 69(3):588–95. 10.1038/sj.ki.5000116 16395250

[34] Munshi VN Saghafian S Cook CB Werner KT Chakkera HA. Comparison of post-transplantation Diabetes Mellitus Incidence and Risk Factors between Kidney and Liver Transplantation Patients. PLoS One (2020) 15(1):e0226873. 10.1371/journal.pone.0226873 31923179 PMC6953760

[35] Hornum M Jørgensen KA Hansen JM Nielsen FT Christensen KB Mathiesen ER New-onset Diabetes Mellitus after Kidney Transplantation in Denmark. Cjasn (2010) 5(4):709–16. 10.2215/cjn.05360709 20167685 PMC2849691

[36] Valderhaug TG Jenssen T Hartmann A Midtvedt K Holdaas H Reisæter AV Fasting Plasma Glucose and Glycosylated Hemoglobin in the Screening for Diabetes Mellitus after Renal Transplantation. Transplantation (2009) 88(3):429–34. 10.1097/tp.0b013e3181af1f53 19667949

[37] Miller DR Safford MM Pogach LM. Who Has Diabetes? Best Estimates of Diabetes Prevalence in the Department of Veterans Affairs Based on Computerized Patient Data. Diabetes Care (2004) 27(Suppl. 2):B10–21. 10.2337/diacare.27.suppl_2.b10 15113777

[38] Davidson J Wilkinson A Dantal J Dotta F Haller H Hernández D New-onset Diabetes after Transplantation: 2003 International Consensus Guidelines. Proceedings of an International Expert Panel Meeting. Barcelona, Spain, 19 February 2003. Transplantation (2003) 75(10):SS3–24. 10.1097/01.TP.0000069952.49242.3E 12775942

[39] Ye X Kuo H-T Sampaio MS Jiang Y Bunnapradist S. Risk Factors for Development of New-Onset Diabetes Mellitus after Transplant in Adult Lung Transplant Recipients. Clin Transpl (2011) 25(6):885–91. 10.1111/j.1399-0012.2010.01383.x 21175848

[40] Sharif A Cohney S. Post-transplantation Diabetes-State of the Art. Lancet Diabetes Endocrinol (2016) 4(4):337–49. 10.1016/s2213-8587(15)00387-3 26632096

[41] Lane JT Dagogo-Jack S. Approach to the Patient with New-Onset Diabetes after Transplant (NODAT). J Clin Endocrinol Metab (2011) 96(11):3289–97. 10.1210/jc.2011-0657 22058376

[42] Mizrahi N Braun M Ben Gal T Rosengarten D Kramer MR Grossman A. Post-transplant Diabetes Mellitus: Incidence, Predicting Factors and Outcomes. Endocrine (2020) 69(2):303–9. 10.1007/s12020-020-02339-9 32418071

[43] Cosio FG Pesavento TE Kim S Osei K Henry M Ferguson RM. Patient Survival after Renal Transplantation: IV. Impact of post-transplant Diabetes. Kidney Int (2002) 62(4):1440–6. 10.1111/j.1523-1755.2002.kid582.x 12234317

[44] Yeh H Lin C Li Y-R Yen C-L Lee C-C Chen J-S Temporal Trends of Incident Diabetes Mellitus and Subsequent Outcomes in Patients Receiving Kidney Transplantation: a National Cohort Study in Taiwan. Diabetol Metab Syndr (2020) 12(1):34. 10.1186/s13098-020-00541-3 32368254 PMC7189729

[45] Lim WH Wong G Pilmore HL McDonald SP Chadban SJ. Long-term Outcomes of Kidney Transplantation in People with Type 2 Diabetes: a Population Cohort Study. Lancet Diabetes Endocrinol (2017) 5(1):26–33. 10.1016/s2213-8587(16)30317-5 28010785

[46] Pandey A Forte V Abdallah M Alickaj A Mahmud S Asad S Diabetes Mellitus and the Risk of Cancer. Minerva Endocrinol (2011) 36(3):187–209. 22019750

[47] American Diabetes Association. Diabetes Care in the Hospital, Nursing home, and Skilled Nursing Facility. Diabetes Care (2015) 38:S80–5. 10.2337/dc15-S016 25537715

[48] Kasiske BL Zeier MG Chapman JR Craig JC Ekberg H Garvey CA KDIGO Clinical Practice Guideline for the Care of Kidney Transplant Recipients: a Summary. Kidney Int (2010) 77(4):299–311. 10.1038/ki.2009.377 19847156

[49] Centre of Excellence for Health. Immunity and Infections (CHIP) (2021). Available from: https://chip.dk/Portals/0/files/MATCH/CLASS_Cause%20of%20Death%20List.pdf?ver=2018-10-01-095315-583&timestamp=1538380416865 .

