# Supplementary material for: Posttransplantation Diabetes Mellitus Among Solid Organ Recipients in a Danish Cohort
Source: Transpl Int. 2022 Apr 5;35:10352. doi: 10.3389/ti.2022.10352 (PMC9016119; doi:10.3389/ti.2022.10352)
Supplement: Supplementary file 1 [file DataSheet3.docx]

Supplemental material 3. Number of patients and distribution of EL-PTDM and PTDM diagnostic criteria per transplant type and per time period.
