# Supplementary material for: Posttransplantation Diabetes Mellitus Among Solid Organ Recipients in a Danish Cohort
Source: Transpl Int. 2022 Apr 5;35:10352. doi: 10.3389/ti.2022.10352 (PMC9016119; doi:10.3389/ti.2022.10352)

Supplemental Material 2. Number and % of non-diabetes, pre-transplant diabetes and PTDM overall and per transplant type at each time period. A) Kidney; B) Liver, C) Lung; D) Heart; E) Overall


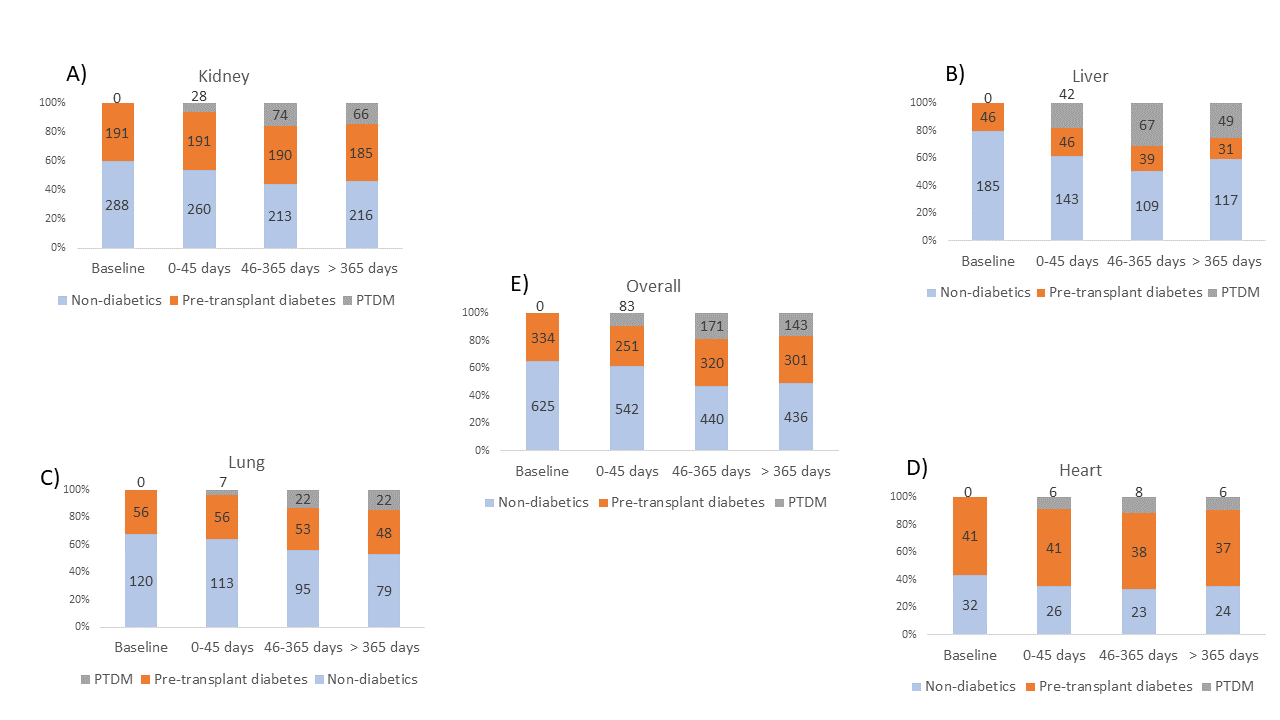

Supplement: Supplementary file 2 [file DataSheet2.docx]
