# Supplementary material for: Posttransplantation Diabetes Mellitus Among Solid Organ Recipients in a Danish Cohort
Source: Transpl Int. 2022 Apr 5;35:10352. doi: 10.3389/ti.2022.10352 (PMC9016119; doi:10.3389/ti.2022.10352)
Supplement: Supplementary file 3 [file DataSheet1.docx]

**Supplemental material 1.**

**Immunosuppressive regimens used by the cohort.**

*Liver transplantation*

Following liver transplantation, a single dose of methylprednisolone 1,000 mg is given intraoperatively. In the days following the transplantation, prednisolone is tapered gradually from 200 mg on day 1 to 30 mg on day 5. For the remaining first month, 20 mg is given daily tapered to 15, 10 and 7.5 mg daily until month 6. For the next 6 months, 5 mg is given daily after which the drug is discontinued. Tacrolimus is given twice daily, aiming at through levels of 10–12 ng/mL in the first month, 8–10 ng/mL in month 2, 7–9 ng/mL in months 3–6, 6–8 ng/mL in months 7–12 and 4–6 ng/mL after 1 year. Mycophenolate mofetil is given twice daily at a dosage of 1,000 mg continuously. In addition, liver-kidney transplant recipients are given a dose of basiliximab 20 mg on day 0 and 4.

*Heart transplantation*

Following heart transplantation, thymoglobulin 1.5 mg/kg for 3 days with 1 g methylprednisolone for 2 days followed by 125 mg for an additional 3 days is used. Prednisone 0.2 mg/kg, tapered to 0.1 mg/kg after 3 months and to zero after 1 year, and mycophenolate mofetil 1–1.5 g × 2 daily continuously. Cyclosporine (or tacrolimus) is used adjusted to through blood levels between 200 and 350 ng/mL (10-15 ng/mL) for the first 6 weeks then reduced to 150–250 ng/mL (8-12 ng/mL) and again to 100 ng/mL (5-8 ng/mL) after one year.

*Lung transplantation*

Following lung transplantation, thymoglobulin 1.5 mg/kg for 3 days with 500 mg methylprednisolone for 1 day followed by 125 mg methylprednisolone each day for 3 days is used. Prednisone 15 mg daily tapered to 5 mg daily over 4 weeks. Azathioprine 1.5 mg/kg from postoperative day 1. Cyclosporine (or tacrolimus) from postoperative day 1 with target range 200–240 ng/mL (10-15 ng/mL) for the first 3 months, 150–200 ng/ml (8-12 ng/mL) for months 4–12, and again to 100-150 ng/mL (5-8 ng/mL) after one year.

*Kidney transplantation*

Following kidney transplantation, typically, induction is given with two doses of 20 mg basiliximab (day 0 and day 4) and methylprednisolone 250 mg (day 0). Maintenance immunosuppression is started at day 1 as triple therapy with tacrolimus (target range 5-10 ng/ml), mycophenolate mofetil (750 mg x 2) and prednisolone (20 mg daily tapered to 5 mg daily the following month).
